# 2,8-dihyroxyadenine (DHA) crystalline nephropathy: A case report 

**DOI:** 10.5414/CNCS111590

**Published:** 2025-03-25

**Authors:** Jawad Iqbal Rather, Mukaresh Fatima, Muzafar Maqsood Wani, Imran Khan, Muzamil Ahmad Wani, Amir Farooq

**Affiliations:** Department of Nephrology, Sher-I-Kashmir Institute of Medical Sciences, Srinagar. India

**Keywords:** 2,8-dihyroxyadenine, APRT, crystalline nephropathy, xanthine oxidase inhibitors

## Abstract

Adenine phosphoribosyltransferase (APRT) deficiency is a rare autosomal disorder with extremely variable presentation. The disease spectrum ranges from completely asymptomatic to 2,8-dihydroxyadenine (DHA) stones to massive deposition of DHA crystals leading to DHA crystalline nephropathy. We report a case of a 45-year-old woman who presented with acute kidney injury and recurrent vomiting. Kidney biopsy revealed precipitation of brown crystals in tubular lumina with acute tubular injury with characteristic birefringence on polarizing light, confirming the unexpected diagnosis of DHA crystalline nephropathy. She was started on a xanthine oxidase inhibitor which resulted in an improvement of kidney function. This case highlights the fact that APRT deficiency can have varied presentations and is an important hereditary cause of crystalline nephropathy.

## Introduction 

Crystalline nephropathies are a unique form of kidney disease. They can be classified by the etiology, for example drug-induced, paraproteinemia-induced, and those associated with inherited disorders. They are often an overlooked cause of kidney disease and are characterized by the finding of crystal deposition predominantly involving the tubulointerstitium of the kidneys [1]. 

Adenine phosphoribosyltransferase (APRT) deficiency is a disease with variable presentation. In addition to causing recurrent kidney stones, the disease can also lead to crystalline nephropathy. Due to its rarity and variable clinical presentation, the diagnosis is often delayed by years after symptom onset, resulting in irretrievable kidney damage in many patients [2, 3]. Here we report the case of a 45-year-old woman presenting with kidney dysfunction and pancytopenia. On kidney biopsy, the patient was diagnosed with 2,8-dihydroxyadenine (DHA) crystalline nephropathy. 

## Case report 

A 45-year-old woman, born to consanguineous parents, with no known comorbidity, presented with complaints of pain in the left upper abdomen, early satiety, and occasional vomiting. There was no history of fever, trauma, jaundice, loose stools, or weight loss. On examination her blood pressure was normal. She had pallor. Abdominal examination revealed moderate splenomegaly. The rest of the examination was unremarkable. Investigations revealed pancytopenia and increased serum urea and creatinine. Investigations are shown in Table 1. 

Peripheral blood film showed pancytopenia with a reticulocyte production index of 1%. Bone marrow examination revealed dimorphic red cell morphology. No other abnormal cells or parasites were seen. Serum vitamin B12 was low, 123 pg/mL (190 – 950 pg/mL). 

Urine examination revealed 1+ protein, no red blood cells or casts, and occasional white blood cells. 24-hour urine protein excretion was 406 mg. In view of kidney dysfunction, antinuclear antibodies, anti-myeloperoxidase antibodies, anti-proteinase 3, and anti-glomerular basement membrane antibodies were done, which were all negative. Serum complement levels were within normal limits. Serum electrophoresis, serum immunofixation, and free light chain assays were negative for paraprotein. Viral serology was negative for hepatitis B, C, and HIV. In view of unexplained kidney dysfunction, a CT-guided percutaneous kidney biopsy was done. 

**Figure 1:**
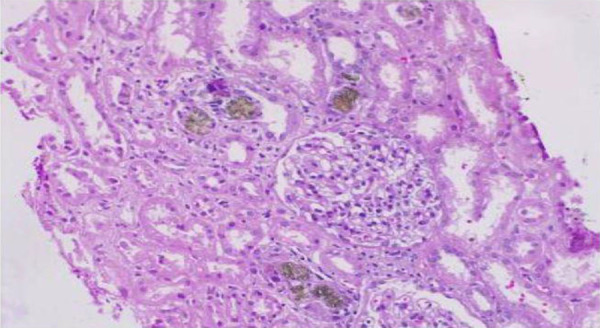
Photomicrograph showing a normal glomerulus with surrounding brown crystals in multiple tubular lumina and focal acute tubular injury suggestive of 2,8-dihydroxyadenine crystalline nephropathy (periodic acid Schiff stain, × 400 magnification).

Light microscopy revealed 8 glomeruli, and none globally sclerosed. One glomerulus showed segmental tuft sclerosis. There was no evidence of any hypercellularity, deposition, or thrombi. Interstitial fibrosis and tubular atrophy (IFTA) was 8 – 10%. Tubules showed focal acute tubular injury. Several tubular lumina showed brown to brownish-green strongly birefringent crystals with focal giant cell reaction. These crystals are stained black with methenamine silver. Immunofluorescence was negative. Urine examination under polarized microscopy revealed characteristic reddish-brown crystals, with characteristic central Maltese cross pattern. In view of characteristic morphological features, the patient was diagnosed as a case of DHA crystalline nephropathy (Figure 1). 

The final diagnosis of 2,8-DHA crystalline nephropathy with concomitant vitamin B12 deficiency was made in this patient, and she was started on febuxostat. Her kidney function improved after the treatment, and her most recent serum creatinine decreased to 2 mg/dL. 

## Discussion 

Cartier et al. [4] in 1974, reported a child with kidney stones caused by total APRT deficiency. His parents were heterozygous for the same mutation. The stone composition revealed a new compound for the first time: DHA. Van Acker et al. [5] subsequently confirmed the autosomal recessive inheritance of APRT deficiency in one family. The family presented with varying manifestations of the disease. The prevalence of DHA stones is particularly high in the Japanese population [6]. 

The APRT enzyme, expressed ubiquitously in all tissues, serves as the sole pathway for the salvage of adenine derived from both dietary sources and polyamine biosynthesis [6]. APRT catalyzes the formation of adenosine monophosphate and inorganic pyrophosphate from adenine and 5-phosphoribosyl-1-pyrophosphate, thus allowing adenine to be detected at very low levels in blood and urine [7]. In patients with APRT deficiency, adenine is converted to 8-hydroxyadenine, which is subsequently converted to DHA by xanthine oxidase [8]. DHA is very insoluble in urine leading to crystallization, which can aggregate, grow, and form stones [9] or deposit in the renal parenchyma, leading to crystalline nephropathy [10]. 

APRT deficiency can present at any age, spanning from infancy to the eighth decade of life [3]. The disease is diagnosed late because it may remain asymptomatic for many years. Moreover, the lack of clinical suspicion, even in patients who have nephrolithiasis, further causes delay in diagnosis. In the context of familial screening, it is not uncommon to identify individuals with complete APRT deficiency who remain entirely asymptomatic, even into adulthood [11]. True to its heterogeneous clinical presentation, our patient remained asymptomatic until 45 years of age. There was no personal or family history of urolithiasis. DHA nephropathy can present as an acute disease, leading to rapid renal failure within days or weeks. However, it more commonly develops gradually, causing a progressive decline in renal function over several years. Dehydration can trigger acute renal failure by causing urine supersaturation and precipitation of DHA, leading to acute kidney injury. If left undiagnosed, this can frequently progress to chronic kidney disease [12]. Our patient who was previously asymptomatic presented with acute kidney injury. We hypothesize that recurrent vomiting lead to dehydration in our patient which caused DHA crystallization leading to kidney dysfunction. 

The diagnosis is primarily based on the identification of DHA. Stones, if available, should be analyzed by infrared spectroscopy which detects DHA crystals in all cases. Crystalluria examination using light and polarizing microscopy remains a valuable and cost-effective method for identifying DHA crystals [13]. This technique exhibits high sensitivity and specificity, enabling the detection of homozygotes in 100% of cases. [12]. Biochemical analysis of stones is not helpful in differentiating uric acid stones from DHA stones. Moreover, both types of stones are radiolucent. Unusually, this disease may be diagnosed unexpectedly on kidney biopsy, as seen in our case. The kidney biopsy shows a variable degree of acute tubular injury and brown crystals in the lumen. Further characterization of these crystals can be done by Fourier infrared transform microscopy and polarizing light [14]. Given the pathognomonic nature of DHA stones or crystals in APRT deficiency, measuring APRT activity in erythrocyte lysates is not strictly necessary but can be a valuable diagnostic tool when available [15]. Mutations located in the promoter region or large deletions within a single allele can lead to a significant proportion (approximately 10%) of mutations being missed, even when the entire coding region and intron/exon junctions of the *APRT* gene are sequenced [3]. 

The treatment of APRT deficiency primarily relies on allopurinol therapy, which inhibits xanthine oxidase. In patients with DHA nephropathy, allopurinol therapy often leads to stabilization or improvement of renal function and can prevent recurrence after renal transplantation [11]. The chances of renal function recovery largely depend on the extent of chronic changes when treatment is started. All patients with complete APRT deficiency, even if they have no symptoms, should receive a xanthine oxidase inhibitor, given the risk of DHA nephropathy. High-purine diets should be avoided and patients should maintain high fluid intake. DHA crystals remain insoluble even up to a pH of 8.5, hence urinary alkalization is not helpful [7]. Our patient was started on allopurinol and vitamin B12 supplementation. She is currently on our follow-up. Her creatinine improved to 2 mg/dL, and pancytopenia resolved. In addition, screening of siblings was advised. 

Readers are further referred to a comprehensive review on crystalline nephropathies by Perazella and Herlitz [16]. 

## Conclusion 

APRT deficiency can have varied presentations, ranging from asymptomatic to recurrent urolithiasis to massive precipitation of DHA crystals in the kidneys. APRT deficiency must be suspected in all cases of nephrolithiasis in children, recurrent nephrolithiasis (particularly if the stones are radiolucent), and nephrolithiasis associated with unexplained kidney dysfunction. Wherever possible, a comprehensive stone analysis should be performed. Crystalluria can be useful, especially when a stone isn’t available for analysis. Similarly, an APRT activity assay is helpful when no stone is available and crystalluria cannot be examined in a patient, such as in cases of anuria, particularly for patients undergoing a renal transplant. Prompt treatment with allopurinol or other xanthine oxidase inhibitors is the cornerstone of the therapy. 

## Authors’ contributions 

Jawad Iqbal Rather: Writing the manuscript, corresponding author, was directly involved with the index patient care. 

Mukaresh Fatima: Writing the manuscript, involved in the index patient care. 

Muzafar Maqsood Wani: Critically reviewing the manuscript, supervising the management of the patient. 

Imran Khan: Writing the manuscript, involved in the index patient care. 

Muzamil Ahmad Wani: Writing the manuscript, involved in the index patient care. 

Amir Farooq: Writing the manuscript, involved in the index patient care. 

All authors take responsibility and accept accountability for the overall work. 

## Funding 

None. 

## Conflict of interest 

None. 

**Table 1.** Table showing various investigations of the patient.

**Parameter**	**Value**	**Reference values**
Hemoglobin g/dL	8.3	12 – 16
Total leukocyte count 10*3/uL	3.7	4 – 11
Platelet 10*5/uL	0.8	1.5 – 4.5
Mean corpuscular volume	103	78 – 95
Urea mg/dL	107	10 – 45
Creatinine mg/dL	5.8	0.5 – 1.5
Urine acid mg/dL	7.2	3.5 – 7.0
Lactate dehydrogenase U/L	246	25 – 248
Creatine phosphokinase U/L	44	55 – 170
Calcium mg/dL	8.2	8.5 – 10.5
Phosphate mg/dL	3.8	2.5 – 4.5
Sodium mmol/L	139	135 – 145
Potassium mmol/L	3.82	3.5 – 5.5
Chloride mmol/L	115	95 – 105
Bicarbonate mmol/L	16.2	22 – 29
Anion gap	9	8 – 12
Bilirubin mg/dL	0.9	0.3 – 1.5
AST U/L	14	0 – 45
ALT U/L	9	0 – 45
ALP U/L	63	30 – 141
Total protein mg/dL	6.3	5.5 – 8.5
Albumin mg/dL	3.7	3.5 – 5.2

AST = aspartate transaminase; ALT = alanine transaminase; ALT = alkaline phosphatase.
